# Protein Structure Determination by Racemic and Quasi‐Racemic X‐Ray Crystallography

**DOI:** 10.1002/cbic.202500950

**Published:** 2026-02-23

**Authors:** Stephen B. H. Kent

**Affiliations:** ^1^ Department of Chemistry Department of Biochemistry & Molecular Biology The University of Chicago Chicago Illinois USA

**Keywords:** chemical ligation, chemical protein synthesis, mirror image proteins, protein structure, X‐ray crystallography

## Abstract

This perspective essay recounts fundamental aspects of two forms of racemic protein crystallography, techniques that significantly enhance the success rate for determining protein molecular structures by X‐ray diffraction. Crystallization from a racemic mixture of protein enantiomers, i.e., the natural chirality L‐protein and its D‐protein enantiomer, gives highly ordered centrosymmetric crystals with a success rate much greater than for the L‐protein alone and even facilitates the crystallization of L‐protein molecules proven to be recalcitrant to crystallization by conventional methods. X‐ray reflections from such centrosymmetric racemic protein crystals have quantized phases, greatly simplifying solution of protein structures by direct methods and giving high‐quality electron density maps. Quasi‐racemic mixtures of protein isomorphs (proteins with mirror image *shapes* that are not true chemical enantiomers) also strongly facilitate the formation of diffraction‐quality crystals. D‐protein molecules are prepared by total synthesis based on modern chemical ligation methods. Examples selected from the literature will be highlighted to illustrate the application and utility of racemic crystallography for the elucidation of protein structures.

## Graphical abstract

Racemic mixtures of protein enantiomers facilitate formation of diffraction‐quality crystals in centrosymmetric space groups that have quantized phases which simplify structure solution. Quasi‐racemic mixtures of a protein and its mirror image isomorph similarly facilitate formation of highly ordered crystals. Racemic and quasi‐racemic X‐ray crystallography enable elucidation of structures of recalcitrant proteins and protein complexes.

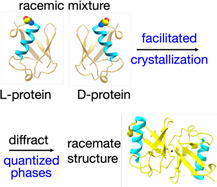

## 1 Introduction

X‐ray crystallography is by far the most successful method for the experimental determination of the structures of protein molecules. To date, 200,000 protein molecule X‐ray structures have been deposited in the Protein Data Bank, where they constitute 90% of all experimental protein structures [1]. Typically, in order to experimentally determine a protein's molecular structure, the target protein is overexpressed in microorganisms such as *E. coli*or yeast engineered by recombinant DNA techniques, and isolated and purified prior to crystallization. To be useful for diffraction studies, the resulting protein crystals must be “diffraction quality,” regular, and free of imperfections. Once a suitable crystal of a protein molecule has been obtained, diffraction data are collected using a synchrotron X‐ray source. The protein crystal is rotated in a monochromatic X‐ray beam, and the intensity and position of the diffracted beams (termed “reflections”) are digitally recorded at each position of the protein crystal. These data are combined computationally into a three‐dimensional dataset [2].

**SCHEME 1:**
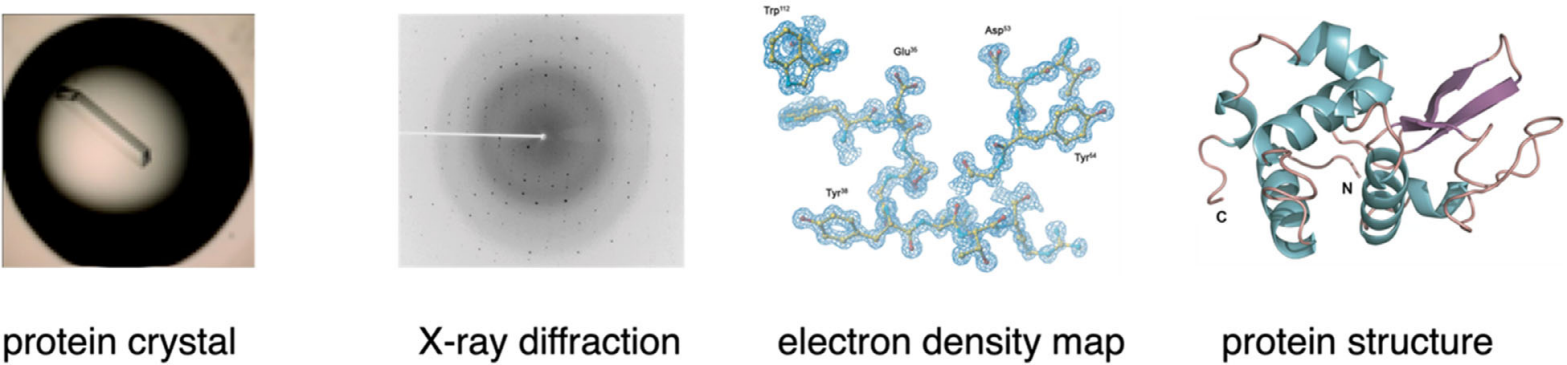
Protein X‐ray crystallography.

The first step in interpreting the diffraction data is to index the reflections in order to determine the “space group” symmetry of the crystal packing.^1^ Mathematicians have shown that there are exactly 230 different ways to form a regular array of objects in three dimensions [3]. As will become significant later in this essay, just 65 space groups are accessible to chiral entities such as protein molecules.^2^ In order to calculate an electron density map from X‐ray diffraction data, both the intensity and phase of each reflection must be known. Phases are not measured directly in the collection of diffraction data, and other means must be used to determine a phase for each reflection. Historically, this was known as the “phase problem” and was one of the major challenges for determination of protein crystal structures by X‐ray diffraction. Current methods for obtaining phase information include multiple wavelength anomalous dispersion (“MAD”) phasing, computationally by “molecular replacement” using initial phases from a closely related protein structure, and “direct” *ab initio* computational methods [4]. Once a set of phases has been obtained, an electron density map can be calculated and a molecular model is then built in three dimensions to fit the observed electron density, as shown in Scheme 1.

Protein X‐ray crystallography is widely used. Currently ~10,000 new X‐ray diffraction protein structures are added to the Protein Data Bank each year [1]. Despite this success, protein crystallography has an “Achilles heel”: the difficulty of obtaining diffraction‐quality crystals from even high purity protein samples. The world‐wide Structural Genomics programs of the early 2000s quantitatively documented this problem: of more than 47,000 purified globular protein molecules, only 16% gave diffraction‐quality crystals—a failure rate greater than 80% (Table 1).

**TABLE 1:** Crystallization statistics for globular protein molecules. Screenshot from Structural Genomics (Phase 2) website. Blue highlighting added.

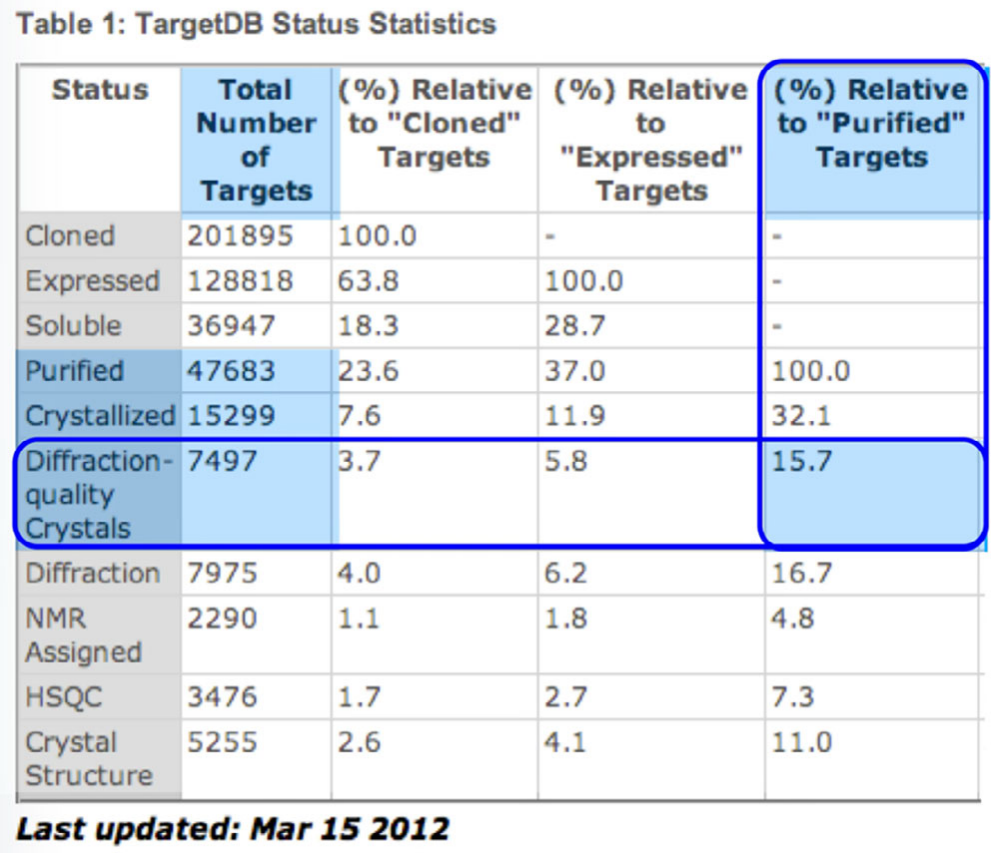

## 2 Protein Space Groups

Proteins that form diffraction‐quality crystals do so in a very limited subset of the 230 possible space groups. Proteins are chiral entities and thus must crystallize in one of the 65 chiral space groups that do not require operations such as inversion or reflection. A single space group, P2_1_2_1_2_1_ is by far the most commonly observed space group for protein crystals, followed by space groups P2_1_ and C2. Together, these three space groups account for more than 50% of observed L‐protein crystal symmetries. A dozen other space groups account for essentially all the other symmetries in crystals of L‐protein molecules used in X‐ray crystallography [5]. In 1995, Wukovitz and Yeates published a theoretical model that accounts for the preference of protein molecules to crystallize predominantly in space group P2_1_2_1_2_1_ and for the relative preferences observed for other protein space groups [6]. Protein crystals contain large amounts of solvent water, often 50% or more, and consist of regular networks of well‐solvated protein molecules in contact with each other. The number of ways that such a protein network can be formed is called “dimensionality” and is a property of the space group symmetry. Wukovitz and Yeates postulated that dimensionality values could be used to rank the space group propensities of protein molecule crystals. Each integral dimensionality value step represents almost an order‐of‐magnitude difference in ease of crystallization, and the predicted rankings are in excellent agreement with the observed space group propensities for L‐globular protein molecules.

The 165 *achiral* space groups are accessible only to *achiral* objects: in the case of a protein molecule, that would consist of a pair of enantiomeric protein molecules suitably arranged and treated as a single achiral entity. Because of the unique, high dimensionality value of a the centrosymmetric^3^ space group, P1<bar>, and the high dimensionality values of the centrosymmetric space groups P21/c and C2/c that have the same D values as the highly favored L‐protein space group P2_1_2_1_2_1_, Wukovitz and Yeates predicted that *racemic protein mixtures would crystallize much more readily* than natural L‐proteins alone, and that the most common racemic protein space group would be P1<bar> [6].

Earlier, in a letter to Nature in1989, the prominent mathematician‐crystallographer Alan Mackay had pointed out that for centrosymmetric racemic protein crystals the X‐ray diffraction pattern would be greatly simplified, because off‐diagonal reflections cancel one another, and the phases of the remaining reflections are obligately related by differences of pi‐radians [7]. For example, in the space group P1<bar>, all reflections obligately have phases of exactly zero or pi radians (0° or 180°). This quantized phase phenomenon could greatly simplify solving the phase problem by direct computational methods. Furthermore, such precise phases can give higher‐quality electron density maps [8, 9].

*Enhanced ease of crystallization of racemic protein mixtures together with the quantized phases of reflections from centrosymmetric racemic protein crystals provide strong incentives to be able to obtain mirror image protein molecules for structure determination by X‐ray crystallography.*

## 3 Mirror Image Proteins

**FIGURE 1:**
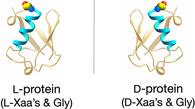
Mirror image protein molecules, illustrated by cartoon representations of ubiquitin.

Natural protein molecules are homochiral. All genetically encoded, ribosomally translated proteins contain linear polypeptide chains consisting of chiral L‐amino acids and the achiral amino acid glycine [10]. Anfinsen's “thermodynamic hypothesis” of protein folding, for which he shared the 1973 Nobel Prize in Chemistry, states that the folded structure of a protein molecule under physiological conditions is determined by the amino acid sequence of its polypeptide chain [11]. Ancillary factors may be involved to *facilitate* folding, usually by preventing misfolding and aggregation, but they do not affect the final, thermodynamically favored, folded structure of the protein molecule's polypeptide chain. The Anfinsen hypothesis implies (but, to my knowledge, was never explicitly stated) that a corresponding polypeptide chain of identical sequence made up of D‐amino acids and glycine will fold to give a D‐protein molecule with folded tertiary/quaternary molecular structure the *mirror image* of the natural L‐protein (Figure 1).

*D‐Proteins cannot be prepared by microbial expression; D‐proteins can only be prepared by total chemical synthesis.*

## 4 Chemical Protein Synthesis

**SCHEME 2:**
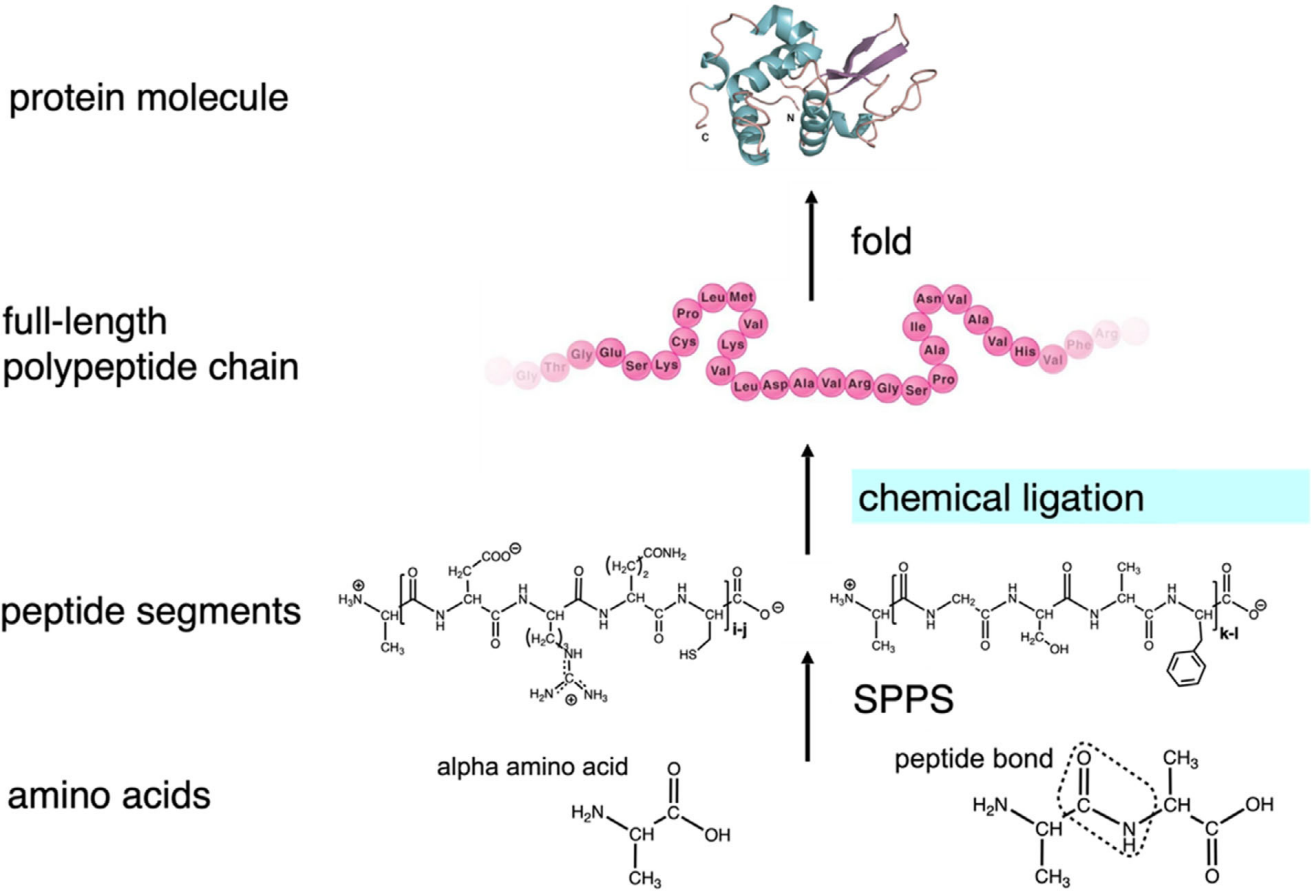
Chemical protein synthesis. Peptide segments encompassing the polypeptide chain of the target protein are made by SPPS. The segments are condensed in convergent fashion by chemical ligation, to give the full‐length polypeptide chain, which is folded to give the synthetic protein molecule.

Convergent chemical ligation of moderately sized, unprotected synthetic peptide segments prepared by solid phase peptide synthesis (SPPS), combined with isolation and purification of intermediate products, enables the straightforward preparation of up to ~150 amino acid protein domains^4^ and similarly sized protein molecules, as outlined in (Scheme 2) [12]. Domain‐sized polypeptide chains can be further condensed by chemical ligation to give the full‐length polypeptide chains found in median ~30‐kDa‐sized proteins [13]. In accord with Anfinsen's thermodynamic hypothesis of protein folding, chemically synthesized full‐length polypeptide chains fold efficiently under well‐established in vitro conditions to give synthetic protein molecules of defined tertiary and quaternary structure. Homogeneity of the chemically synthesized protein molecule is verified by high‐resolution analytical methods based on separation principles *distinct from those used in its purification* [14]. The molecular structure of the synthetic protein, including its covalent chemical structure and amino acid sequence, and its “tertiary” folded structure, can be verified by high‐resolution X‐ray crystallography [15]. More than a thousand protein molecules have been prepared by total chemical synthesis using chemical ligation methods [16] including mirror image enzyme molecules and a variety of other mirror image proteins with masses ranging from <4–100 kDa [17].

## 5 Racemic Protein Crystallography

After the introduction of Fmoc chemistry SPPS in the 1980s, peptide chains containing up to ~40 amino acid residues became accessible in research laboratories by automated stepwise chemical synthesis [18, 19]. Effective purification and analytical characterization gave synthetic peptides as homogeneous molecular species of defined chemical structure. Automated SPPS enabled the total chemical synthesis of small globular protein molecules in both their natural L‐protein form and as mirror image D‐proteins.

**FIGURE 2:**
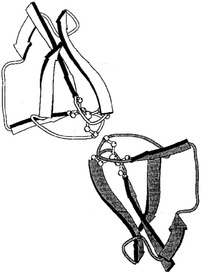
(Left) Crystal structure of racemic rubredoxin. L‐rubredoxin (white); D‐rubredoxin (gray‐black).

In 1993, Zawadzke and Berg reported the first X‐ray diffraction studies of a racemic protein crystal [8]. SPPS was used to make the 45‐amino acid residue polypeptide chain of the iron‐binding protein rubredoxin in D‐amino acid and L‐amino acid forms. Circular dichroism spectra of the folded, Fe^3+^‐containing protein enantiomers showed that they were mirror images of one another. Crystals were obtained from a racemic mixture of the rubredoxin enantiomers under conditions similar to those used to crystalize the synthetic L‐rubredoxin protein. Analysis of X‐ray diffraction patterns revealed that the racemic crystals were centrosymmetric and indexed in space group P1<bar>. The structure of the protein racemate was solved by molecular replacement using the already known structure of L‐rubredoxin and its digitally inverted form, D‐rubredoxin (Figure 2). As expected for the precise, quantized phases from a centrosymmetric space group P1<bar>, the resulting electron density map was very well defined [8].

*This study showed that centrosymmetric protein crystals could be obtained from a mixture of protein enantiomers and pioneered the field of racemic protein crystallography.*

In 1994, chemical synthesis and racemic crystallization of enantiomeric forms of the intensely sweet protein monellin were reported [20]. The monellin protein molecule is a noncovalent heterodimer made up of a 44‐residue A‐chain and a 40‐residue B chain. Each of the two different chains was synthesized by stepwise SPPS in both D‐ and L‐amino acid polypeptide forms. After separately folding the pairs of same chirality peptide chains to give heterodimeric monellin molecules, circular dichroism (CD) spectra showed D‐monellin to be the mirror image of L‐monellin. Racemic crystals were obtained from a solution containing equal amounts of the monellin protein enantiomers. The X‐ray structure of the monellin racemate was reported some years later [21]. Diffraction intensity statistics showed the crystals to be centrosymmetric, and the diffraction data were indexed in space group P1<bar>. The structure of the racemate was solved by molecular replacement, using the already known structure of L‐monellin as search model, and refined in space group P1<bar>. The diffraction data were also refined in space group P1, in which the folded mirror image polypeptide models were independently fitted to the electron density. Intriguingly, the results revealed small but significant differences in the structures of D‐monellin and L‐monellin, suggesting that the mirror image proteins may not be true enantiomers in the racemic crystal.^5^

## 6 Determination of Novel Protein Structures by Racemic Crystallography

In 2008, the first determination of the previously unknown structure of a protein molecule by racemic protein crystallography was reported for snow flea antifreeze protein (sfAFP) [22]. The predicted 81‐residue amino acid sequence of sfAFP is glycine rich and characterized by a repetitive Gly‐Xaa‐Yaa motif, the presence of several proline residues, and contains four Cys residues that form two intramolecular disulfides. The sfAFP polypeptide chain was synthesized in both D‐ and L‐forms by native chemical ligation condensation of four unprotected peptides. After purification, the enantiomeric polypeptide chains were separately folded under standard redox conditions to give the mirror image proteins D‐sfAFP and L‐sfAFP, each of which contained two disulfide bonds.^6^

Crystal formation from a racemic solution containing equal amounts of the chemically synthesized proteins D‐sfAFP and L‐sfAFP occurred much more readily than for L‐sfAFP alone. In a matter of days, a racemic protein solution containing equal amounts of D‐sfAFP and L‐sfAFP gave crystals in ~50% of the 194 conditions initially explored using commercially available Hampton Index crystal screens. This was in marked contrast to what had been observed in attempts over a 6‐month period to obtain diffraction‐quality crystals of L‐sfAFP alone. Synchrotron X‐ray diffraction data were collected from a crystal of the sfAFP racemate to a resolution of 1.0 Å and were indexed in the centrosymmetric space group is: *p*<1bar>. In the absence of phase data, the structure could not be solved by molecular replacement: at the time this work was done, there were no known globular protein homologs of sfAFP. Direct methods also failed.

**FIGURE 3:**
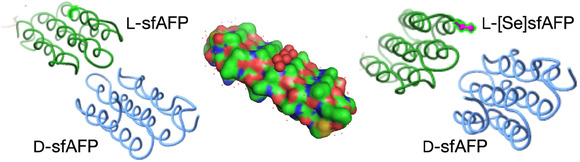
Snow flea anti‐freeze protein. Right: Structure of the sfAFP quasi‐racemate (PDB code 3BOG). Left: Structure of the sfAFP true racemate (PDB code 3BOI). Center: the sfAFP protein molecule, showing its compact folded structure. A set of six bound water molecules is highlighted as red spheres.

It was decided to use multiple wavelength anomalous dispersion (MAD)phasing, but extensive efforts to grow crystals of a synthetic L‐[Se]sfAFP protein met with no success. Facile production of crystals from a *quasi*‐racemic mixture of D‐sfAFP and L‐[Se]sfAFP, a chemical protein analog that contained an additional ‐SeCH2‐ moiety in the side chain of a single previously Asn residue and thus differs slightly from the true enantiomer, was critical for obtaining diffraction‐quality crystals. Synchrotron X‐ray diffraction data were collected to a resolution of 1.2 Å from crystals of the D‐sfAFP and L‐[Se]sfAFP quasi‐racemate, which had identical unit cell parameters to the true racemate. The structure was solved in pseudo‐centrosymmetric space group P1 using MAD data collected at three wavelengths (Figure 3).^7^ The snow flea antifreeze protein molecule was found to have a tertiary structure consisting solely of stacked PPII helices. At that time, this was a previously unknown, but very intriguing, globular protein topology [23].

**SCHEME 3:**
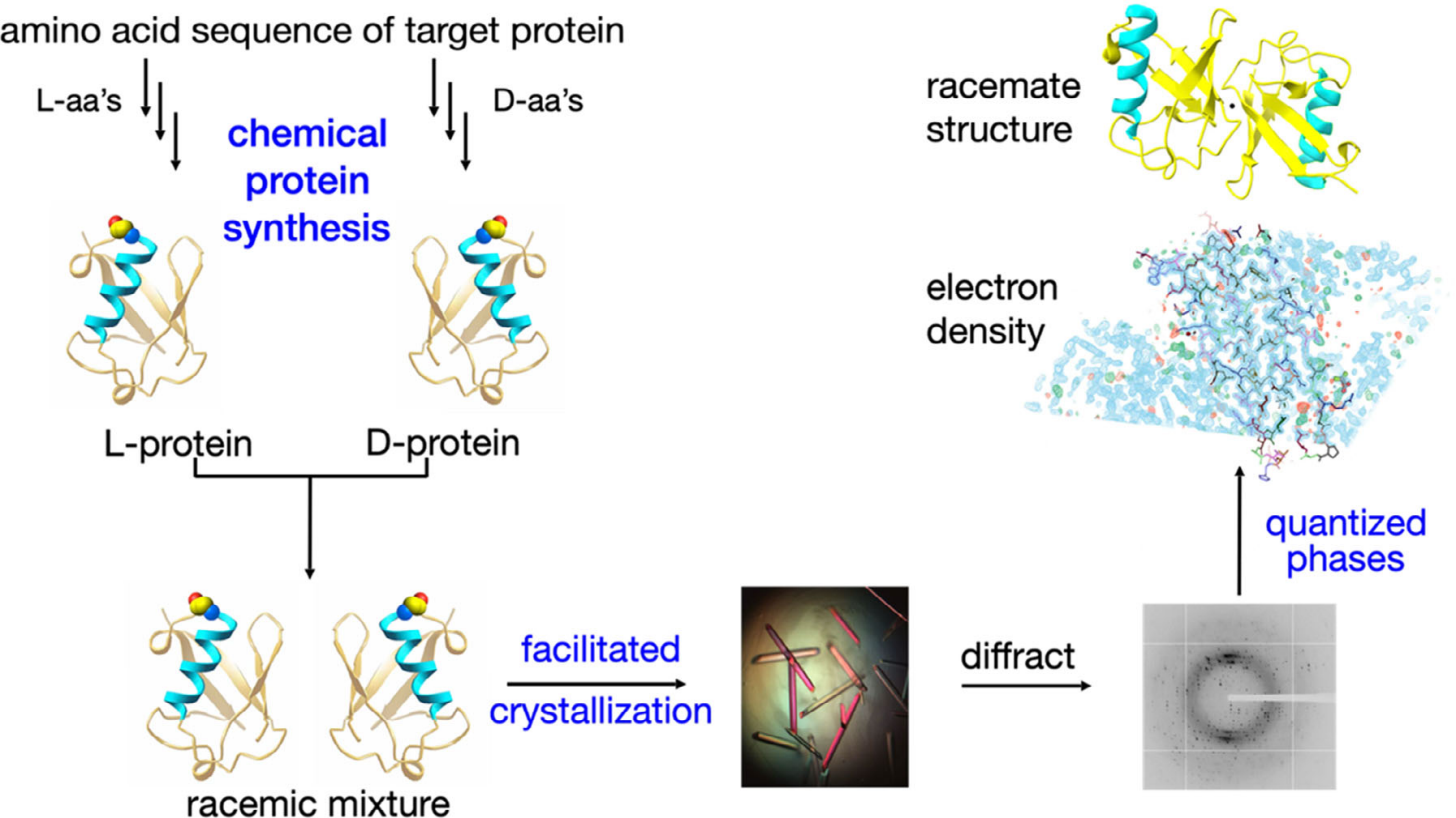
Racemic protein crystallography. D‐protein and L‐protein enantiomers are prepared by total chemical synthesis and co‐crystallized as a 1:1 molar racemic mixture. X‐ray diffraction data are collected from the racemic crystals and, after phasing, are processed to give a high resolution electron density map, to which the mirror image proteins structures are fitted as the centrosymmetric racemate.

Publication of the snow flea antifreeze protein structure determination inspired research groups throughout the world to use racemic crystallography to determine protein structures (Scheme 3).

Protein molecules of natural L‐chirality that had failed to give diffraction‐quality crystals in extensive trials were readily crystalized from racemic protein mixtures. Notably, the protein ubiquitin, a notoriously recalcitrant protein that in multiple laboratories had been shown to be difficult or impossible to crystallize without seeding, readily gave diffraction‐quality racemic crystals overnight in standard Hampton Index screens [24]. In the Author's own laboratory, diffraction‐quality racemic crystals were readily obtained for 17 of 20 different protein molecules, most of which were known to be difficult or impossible to crystallize as the L‐protein alone. That represented a crystallization success rate greater than 80%, which is in marked contrast to the less than 20% success rate of conventional methods observed for obtaining diffraction‐quality crystals from globular L‐proteins.

## 7 Case Studies

The following sections contain selected “Case Studies” chosen to illustrate key features of protein structure determination by racemic and quasi‐racemic crystallography.

### 7.1 Facilitated Crystallization, etc. Snow Flea Antfreeze Protein

Determination of the structure of the sf AFP protein was a milestone in the elucidation of protein structures by racemic crystallography. It experimentally validated the predicted more facile crystallization of a racemic protein mixture compared to the L‐protein alone. In addition, it introduced the use of *quasi*‐racemic pairs of protein molecules to facilitate both crystallization of recalcitrant proteins and the determination of phases for solving quasi‐racemic structures by multiple wavelength anomalous dispersion [22].

### 7.2 Racemic Protein Structure Solution by Direct Methods Venom Protein NmBTx1

Attempts to obtain crystals of the small protein BmBKTx1 were reported to have failed. BmBKTx1 is a 31‐residue protein molecule crosslinked by three disulfide bonds. A racemic mixture of the chemically synthesized proteins D‐BmBKTx1 and L‐BmBKTx1 prepared by stepwise SPPS readily crystallized in the tetragonal centrosymmetric space‐group I41/a, as shown by synchrotron diffraction data collected to 1.1 Å resolution. The structure was solved using direct methods (PDB code 3E8Y) [25].

### 7.3 Racemic Protein Crystals With Exceptionally Low Solvent Content (13%–15%)

#### 7.3.1 Plectasin

Racemic crystallography of chemically synthesized plectasin enantiomers was used to determine the X‐ray structure of the small protein plectasin by direct methods (PDB code 3E7R). Plectasin is a 40‐residue protein molecule containing three disulfide bonds. It is interesting to note that the racemic plectasin crystals had an exceptionally low solvent content (13%–15%), significantly lower than found for the L‐plectasin crystals (40%–42%). The solvent content of protein crystals usually falls in the range 27%–65% [26].

In an Editorial Commentary accompanying publication of the plectasin racemate paper in the journal Protein Science, the eminent crystallographer Brian Matthews explained the potential advantages of racemic protein crystallography which encompass facilitated crystallization, quantized phases that simplify determination of structures by direct methods, and higher‐quality electron density maps [27].

### 7.4 Structure of a Novel Protein Analog by Quasi‐Racemic X‐Ray Crystallography

#### 7.4.1 Crambin Topologue

The plant seed protein crambin has been widely used as a model for protein crystallography methods. Crambin has a polypeptide chain of 46 amino acid residues that contains six Cys residues which form three disulfide bonds in the native protein molecule. A salt bridge between the guanidinium of the Arg^10^ side chain and the alpha carboxylate of Asn^46^ at the C terminus of the polypeptide chain is important for the folding and stability of the crambin protein molecule. Total chemical synthesis from three synthetic peptide segments was used to make a 46‐residue linear‐loop polypeptide in which a *covalent bond* replaced the salt bridge between the side chain of Arg^10^ and the alpha carboxylate of the Asp^46^ C‐terminal residue. Under the same redox conditions used to fold the linear crambin polypeptide chain, the synthetic linear‐loop polypeptide chain folded quantitatively within 30 min to give a topological analog (“topologue”) of the crambin protein molecule. Mass measurement showed that the crambin topologue protein contained three disulfide bonds.

**FIGURE 4:**
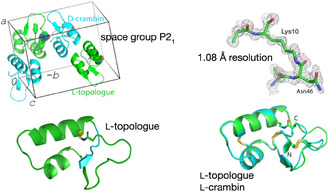
Quasi‐racemic crystal structure of the L‐crambin topological analog (“topologue”) with D‐crambin (PDB code 3UE7). Top left: Crystal unit cell. Bottom left: Structure of the L‐crambin topologue protein molecule, in which a macrocyclic polypeptide chain is cyclized by covalent attachment of the C‐terminal residue to the side chain of Lys^10^. Several N‐terminal amino acid residues penetrate the formed macrolactam ring. Top right: Close up of the amide bond between the side chain of Lys^10^ and the α‐carboxyl of Asp^46^. Bottom right: Comparison of the crambin topologue structure to that of native crambin, by super‐position of the L‐protein topologue on the digitally inverted structure of D‐crambin.

The structure of the crambin topologue protein was determined by quasi‐racemic protein X‐ray crystallography. Crystallization trials of the L‐sfAFP topologue protein over several months under a wide range of conditions failed to give crystals. In dramatic contrast to the failure to obtain crystals under standard screening conditions with the topologue protein alone, a solution that contained equal amounts of the L‐topologue and D‐crambin produced crystals within 4 days under 40%–50% of the conditions examined in a standard Hampton Research Index screen. Synchrotron diffraction data were collected to a resolution of 1.08 Angstrom. The quasi‐racemate diffraction data were indexed in the monoclinic space group P2_1_ and the structure was solved by molecular replacement (PDB code 3UE7). The asymmetric unit had two molecules, D‐crambin and L‐topologue, arranged in a (pseudo)centrosymmetric fashion. The amide bond between the alpha carboxylate of Asn^46^ and the epsilon amino group of Lys^10^ (replacing Arg^10^) was clearly evident in the electron density map (Figure 4) [28].

This work again illustrates greatly facilitated crystallization of *quasi*‐racemic protein mixtures, as was the case for sfAFP. Apparently, mirror image molecular shapes (“enantiomorphs”) are more important than the precise chemical identity of enantiomers for regular packing in quasi‐centrosymmetric protein arrays, which thus form highly ordered crystals in which the protein enantiomorph pairs are related through a (pseudo)center of inversion. It also demonstrated the utility of quasi‐racemic protein crystallography for determining in a single X‐ray diffraction experiment how the structure of an analog protein is related to the native protein molecule, simply by digital inversion of the reference D‐protein structure determined at the same time. Here, the crambin topologue protein was shown to contain the three native disulfide bonds and to only differ slightly from the structure of native crambin in the conformation of a flexible loop in the C‐terminal region of the polypeptide chain.

### 7.5 Quaternary Structure of a Protein Complex by Racemic Crystallography

#### 7.5.1 Heterochiral L‐VEGF A/D‐Protein Binder Complex

**FIGURE 5:**
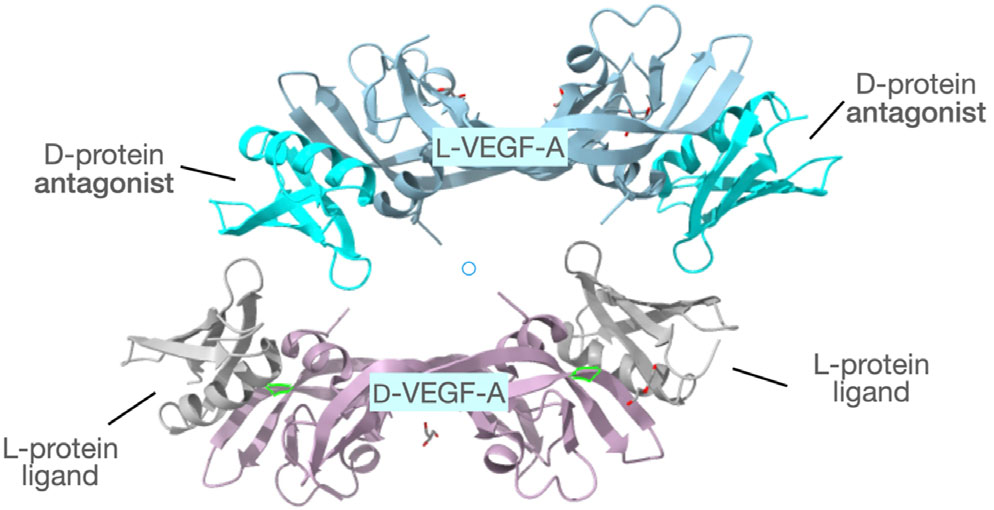
Racemic crystallography of a heterochiral VEGF–binder complex (PDB code 4GLN). Two copies of a 6.5‐kDa D‐protein are bound, one at either end of the L‐VEGF A protein molecule. Similarly, two copies of a 6.5‐kDa L‐protein are bound, one at either end, to the D‐VEGF A protein molecule.

Racemic crystallography was used to determine the structure of a 37‐kDa quaternary protein complex comprised of the 24‐kDa protein L‐VEGF A and two molecules of a 6.4‐kDa D‐protein binder generated by mirror image phage display of variants of the B1 domain of streptococcal protein G (GB1). The L‐VEGF A protein is a covalent homodimer of identical 102 residue domains, each containing three intradomain disulfide bonds, with two inter‐domain disulfides covalently linking the domains. The enantiomeric protein molecules D‐VEGF A and L‐VEGF A, D‐binder protein and L‐binder protein, were prepared by total chemical synthesis. Crystallization trials using a solution containing synthetic L‐VEGF‐A and two molar equivalents of synthetic D‐binder protein failed to give diffraction‐quality crystals. Diffraction‐quality crystals were readily obtained from a solution of chemically synthesized protein molecules that contained one molar equivalent each of L‐VEGF‐A and D‐VEGF‐A, and two molar equivalents each of the L‐protein binder and D‐protein binder molecules. Synchrotron diffraction data were collected to a resolution of 1.6 Å and indexed in the centrosymmetric space group P2_1/n_. The structure of the protein complex was solved by molecular replacement, using inverted and noninverted coordinates of the previously reported crystal structures of VEGF (PDB code: 3QTK) and GB1 (PDB code: 2QMT). The unit cell of the racemic crystal contained 12 chemically synthesized protein molecules: 2 × L‐VEGF A; 2 × D‐VEGF A; 4 × D‐protein binder; 4 × L‐protein binder, and had a structure mass of 74 kDa. As expected from the twofold axis of symmetry of the VEGF A molecule, there were two protein binder molecules bound to each VEGF A protein (Figure 5) [29].

The 37‐kDa protein complex, consisting of 24‐kDa VEGF A and two copies of the 6.5‐kDa D‐protein binder, was by a wide margin the largest protein structure determined by racemic protein crystallography*.* This work demonstrated that racemic crystallization could be used to determine the native quaternary structure of protein complexes.^8^ It was anticipated by a number of years later work which claimed the formation of native tetramers of the short peptide mellitin in racemic crystals as the first example of a racemic structure of quaternary protein complexes [30]. That same paper “suggested” that racemic crystallography could provide insights on native quaternary structures. Indeed it can, as was already been shown by elucidation of the structure of the {VEGF A + D‐binder} protein complex more than 6 years earlier [31].

### 7.6 Facilitated Crystallization of a Class of Recalcitrant Protein Molecules

#### 7.6.1 Cyclic Disulfide‐Rich Peptides

Disulfide‐rich, small protein molecules that have a circularized polypeptide chain are notoriously reluctant to give diffraction‐quality crystals. Racemic mixtures of chemically synthesized enantiomers for three representative cyclic disulfide‐rich peptides containing one, two, and three disulfides, respectively, readily gave crystals that diffracted to better than 1.9 Å. The data were indexed in centrosymmetric space groups and the structures were solved by molecular replacement using NMR structures as search models. Quasi‐racemic mixtures with each of two analogs of the largest cyclic disulfide‐rich peptide, the cyclotide kalata B1 which contained three disulfides, readily gave diffraction‐quality crystals indexed in the pseudo‐centrosymmetric space group P1 and enabled elucidation of the structure of each analog and its reference native structure in the same experiment [32]. More recently, quasi‐racemic crystallography of chemically synthesized molecules was used to elucidate the first crystal structures of the larger “bracelet” cyclotides [33].

### 7.7 Facilitated Crystallization of a Glycoprotein

#### 7.7.1 Glycosylated Ser‐CCL1

Glycoproteins can be difficult to crystallize because of the heterogeneity and flexibility of the glycan moietie(s). The 74‐residue chemokine Ser‐CCL1 contains three disulfide bonds. Glycosylated Ser‐CCL1 was prepared by chemical synthesis using an N‐linked asialo biantennary nonasaccharide glycan moiety of defined covalent structure at Asn^29^. The glycosylated Ser‐CCL1 L‐protein molecule failed to give diffraction‐quality crystals after extensive trials over several months. A quasi‐racemic mixture of chemically synthesized D‐protein Ser‐CCL1 and glycosylated L‐protein Ser‐CCL1 formed crystals within 24 h and gave diffraction‐quality crystals in 2–3 weeks’ time. Synchrotron diffraction data were collected to a resolution of 2.1 Å and the data were indexed in the pseudo‐centrosymmetric space group P1. The quasi‐racemate structure was solved by molecular replacement using the known structure of CCL1 as search model (PDB code 4OIK). In the crystal, the D‐Ser‐CCL1 and the glycosylated L‐protein each form dimers associated by an antiparallel beta‐sheet interface with another molecule of the same chirality. Glycosylation did not affect the structure of the Ser‐CCL1 protein molecule. In the quasi‐racemate crystal, the nonasaccahraide glycan moiety was conformationally disordered—only the GlcNAc residue N‐linked to Asn^29^ of the Ser‐CCL1 protein molecule gave discrete electron density [34].

### 7.8 Facilitated Crystallization of an Extremely Recalcitrant Protein Molecule; Structure Solved by Modified Direct Methods Suggesting Biological Function

#### 7.8.1 Rv1738 from Mycobacterium Tuberculosis

**FIGURE 6:**
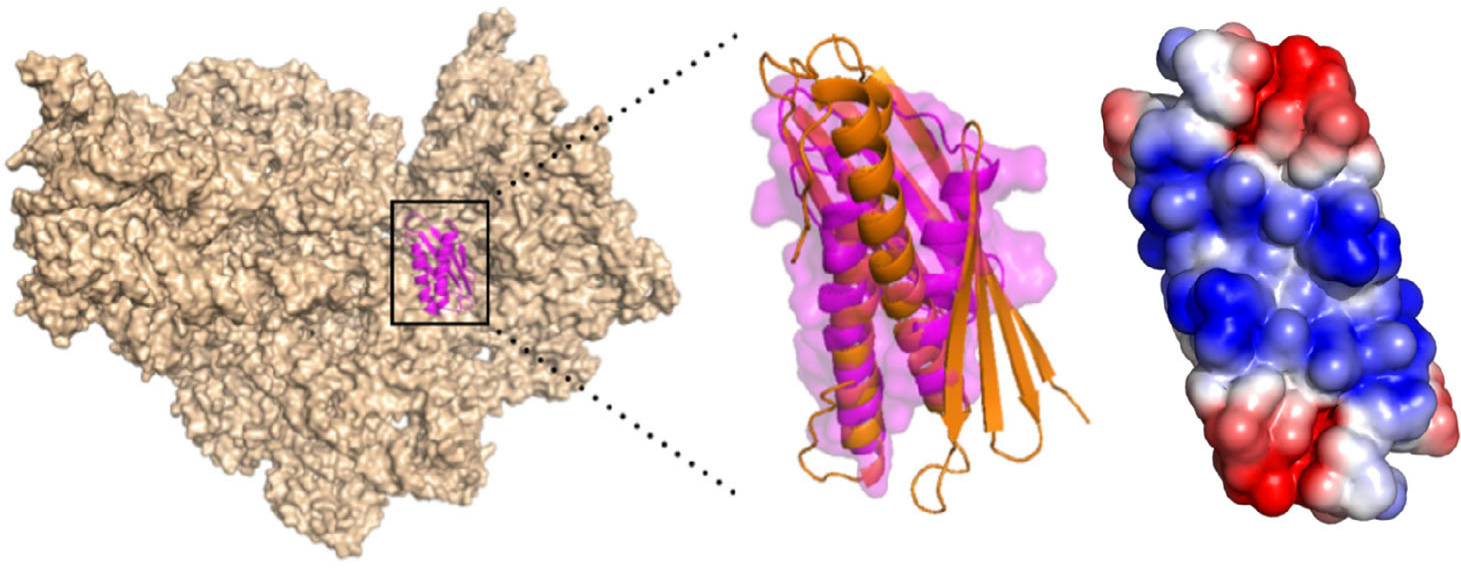
Similarity of Rv1738 and hibernation‐promoting factors. Left: Structure of a hibernation‐promoting factors(HPF) bound to the ribosome (PDB code 4V8I). Middle: Superposition of Rv1738 (orange) and HPF protein (purple). Right: Conserved belt of positive charge on RV1738 [35].

The Rv1738 gene is the most upregulated to produce protein when *Mycobacterium tuberculosis* (M. tb) enters persistent dormancy. For that reason, determination of the structure of the Rv1738 protein molecule was an important objective of the Structural Genomics programs. The predicted protein Rv1738 was readily expressed in *E. coli* using recDNA techniques, but despite numerous attempts over a 3‐year period it was not possible to obtain diffraction‐quality crystals of the Rv1738 L‐protein molecule. The D‐ and L‐amino acid enantiomeric forms of the 94‐residue polypeptide chain of Rv1738 were prepared by chemical synthesis and within a matter of days a racemic mixture gave protein crystals that diffracted to a resolution of 1.5 Å with reflections indexed in the centrosymmetric space group C2/c. The structure was solved by modified direct methods and showed that Rv1738 is a homodimeric protein molecule in which two long *α*‐helixes, one from each monomer, pack in antiparallel fashion and are wrapped by a six‐stranded anti‐parallel *β*‐sheet (Figure 6). A search with the Rv1738 homodimer protein molecule revealed a surprising similarity to a family of stress proteins known as hibernation‐promoting factors (HPFs). This suggests that the functional role of the upregulated Rv1738 protein in M. tb persistent dormancy is to shutdown ribosomal protein synthesis [35].

### 7.9 Structures of Synthetic Di‐Ubq and Tri‐Ubq Isopeptide‐Linked Protein Chains Solved by Quasi‐Racemic Crystallography

#### 7.9.1 Di‐Ubq and Tri‐Ubq Lys^27^ Isopeptide‐Linked Polypeptide Chains

Di‐Ubq and tri‐Ubq chains isopeptide‐linked at Lys^27^ were made by convergent chemical ligation condensation of four and six synthetic peptide segments, respectively. Mirror image forms of the di‐Ubq and tri‐Ubq Lys^27^ isopeptide‐linked chains were prepared in the same fashion using D‐amino acids and glycine; two amino acid substitutions (Met1 to Nle1; Gly to Cys at the isopeptide bond) were used in order to simplify these D‐protein syntheses, giving isomorphous mirror image proteins (i.e., enantiomorphs). Separate quasi‐racemic mixtures consisting of {L‐di‐Ubq + D‐di‐Ubq*} and {L‐tri‐Ubq + D‐tri‐Ubq*} readily formed crystals, which diffracted to 1.6 and 2.1 Å and were indexed in the pseudo‐centrosymmetric space groups C2 and H3, respectively. Both quasi‐racemic structures were solved by molecular replacement (PDB codes 5J8P and 5JBV). This work is a vivid demonstration of the power of facilitated crystallization from quasi‐racemic mixtures of protein enantiomorphs [36].

### 7.10 Quasi‐Racemic Crystallography of Multidomain Homo‐Oligomers by Facilitated Cocrystallization with Multiple Copies of the Corresponding D‐Protein Monomer

#### 7.10.1 Monomer‐Oligomer Quasi‐Racemic Protein Crystallography

**FIGURE 7:**
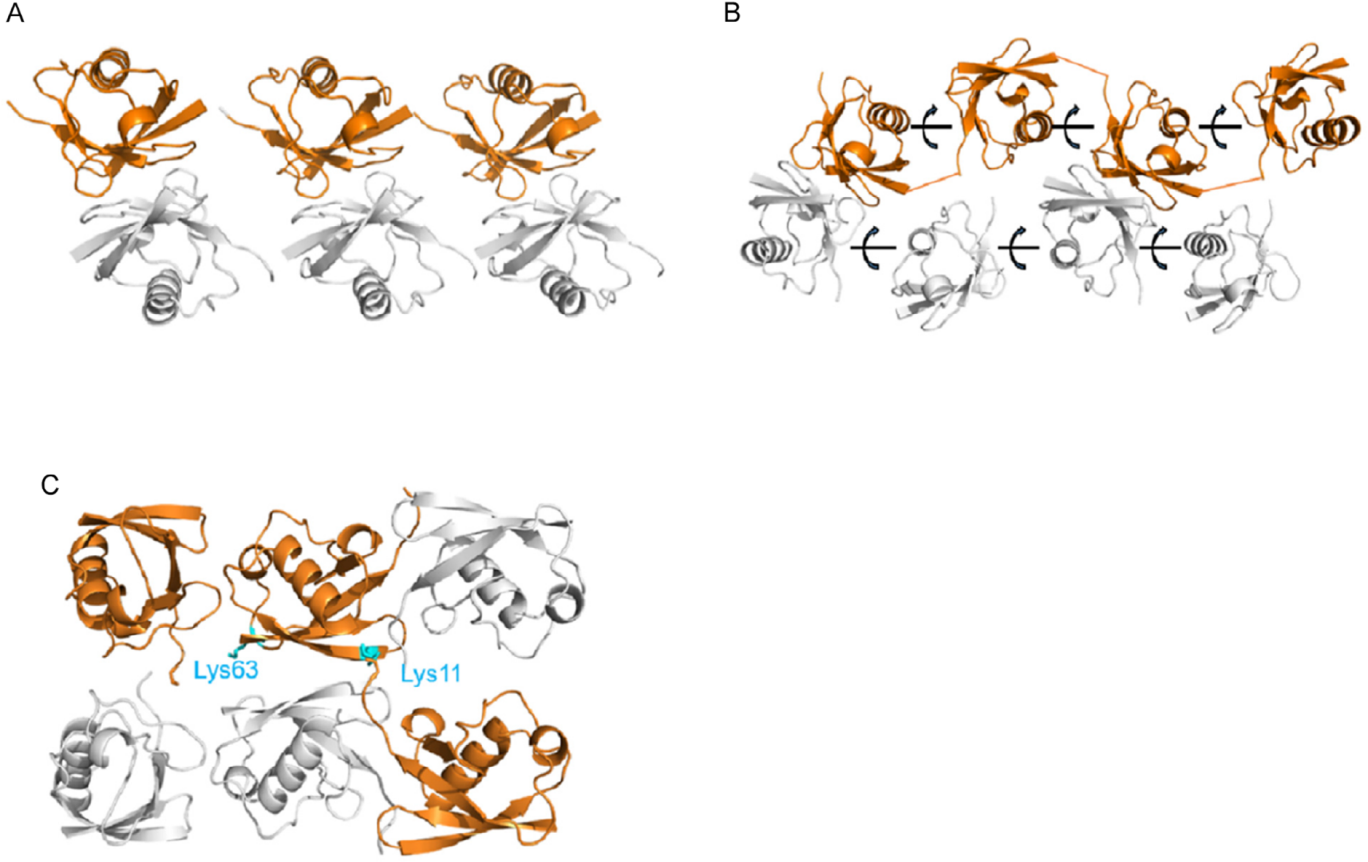
Monomer‐oligomer quasi‐racemate structures [37]. (A) Linear covalent tri‐L‐Ubq and three monomer D‐Ubq molecules (PDB code 3GO7). (B) Linear covalent tetra‐L‐Ubq and four monomer D‐Ubq molecules (5GO8). (C) Covalent K11/K63 branched tri‐Ubq, and three monomer monomer D‐Ubq molecules, arranged to form a pseudo‐centrosymmetric structure (PDB code 5GOK).

X‐ray structures of linear Met^1^‐linked tri‐Ubq, Met^1^‐linked tetra‐Ubq oligomers, and a K11/K63‐branched tri‐Ubq were determined by quasi‐racemic crystallization with multiple copies of D‐ubiquitin (Ubq) monomer. The linear and branched oligo‐Ubq constructs were expressed in *E. coli*, while the K11/K63‐branched tri‐Ubq was prepared by total chemical synthesis; no crystals of these purified L‐protein Ubq oligomers were obtained under exhaustive screening conditions carried out over several months. Quasi‐racemic mixtures consisting of each Ubq oligomer with chemically synthesized monomeric [Nle1]ubiquitin in molar amounts matched to the number of Ubq monomers in the oligo‐Ubq constructs easily and rapidly produced diffraction‐quality crystals of each oligo‐Ubq construct in pseudo‐centrosymmetric space groups (Figure 7). Similar quasi‐racemic {2xD‐monomer + L‐dimer} co‐crystallization was performed on branched ubiquitin dimers isopeptide‐linked at seven different lysine side chain positions, all prepared by chemical protein synthesis. Diffraction‐quality quasi‐racemic crystals of each branched construct were rapidly obtained under multiple conditions. Seven of the ten quasi‐racemic crystals were indexed in space group P1. All but three of the 10 quasi‐racemic crystals were indexed in the pseudo‐centrosymmetric space group P1. X‐ray structures were solved by molecular replacement and showed that in every case the monomeric Ubq molecules had arranged themselves in the crystalline state to form a pseudo‐mirror image of the conformation of the covalent oligomer protein molecule. The quasi‐racemic crystals of the branched di‐Ubq constructs had low solvent contents, ranging from 25% to 35% [37].

### 7.11 Determination of a Novel Protein Fold by Quasi‐Racemic Crystallography Using Radiation Damage Phasing

#### 7.11.1 Antimicrobial Protein Snakin‐1

Snakin is a 63‐residue protein molecule that contains six disulfide bonds which are conserved throughout the GASA/snakin protein superfamily. The first structure of a member of this family of protein molecules was determined by chemical synthesis of the Snakin molecule in both L‐protein and D‐protein forms. Racemic crystals were readily obtained and diffracted to a resolution of 1.6 Å indexed in space group PI <bar>, but attempts to solve the structure by molecular replacement and direct methods failed. The structure was determined using diffraction data at resolutions of 1.5–1.6 Å from quasi‐racemic crystals, which contained {L‐[(4‐iodo)Phe^25^]Snakin + D‐Snakin}, by radiation‐induced damage phasing and revealed the unique disulfide pairings of a novel protein fold which differed from those predicted by computational methods(PDB code 5E5Q) [38].

### 7.12 Quasi‐Racemic Crystallography of Protein Side Chain Diastereomers

#### 7.12.1 L‐allo‐Ser, L‐allo‐Thr ShK Toxin Analogs

Only two of the 20 amino acids normally found in natural protein molecules have a chiral center in their side‐chain: L‐isoleucine (L‐Ile) and L‐threonine (L‐Thr). Inversion of the side chain chiral center gives the diastereomeric amino acids L‐allo‐Ile and L‐allo‐Thr. ShK toxin is a small protein that has two Ile and four Thr residues and which contains three disulfide bonds. Guided by free energy calculations, three side chain diastereomers of the ShK protein were prepared by chemical synthesis: [allo‐Ile^7^]ShK; [allo‐Thr^13^]ShK; and [allo‐Thr^31^]ShK. All three of these protein analogs had folding propensities and thermal stabilities in agreement with calculations. The structures of the three diastereomeric ShK analogs were determined by crystallization from individual quasi‐racemic mixtures, each of which contained one diastereomer and an equal amount of D‐ShK protein.

Solutions containing the protein diastereomers L‐[allo‐Ile^7^]ShK and L‐[allo‐Thr^13^]ShK gave quasi‐racemic crystals that diffracted to resolutions of 1.2 and 0.9 Å respectively. These two quasi‐racemate structures were solved by molecular replacement and showed that both these diastereomers had folded structures closely similar to native ShK (PDB codes 5I5A, 5I5B). However, under multiple conditions, the quasi‐racemic solution of the third diastereomer, [allo‐Thr^31]^ShK, spontaneously resolved to give apparently identical crystals which contained L‐[allo‐Thr^31^]ShK alone and that diffracted to 1.3 Å resolution (PDB code 5I5C). Interestingly, the [allo‐Thr^31^]ShK diastereomer that underwent spontaneous resolution from a quasi‐racemic mixture was significantly less stable than native ShK by both free energy calculations and thermal stability measurements [39].

### 7.13 Quasi‐Racemic Crystallography to Determine the Structure of a Novel Analog Protein

#### 7.13.1 Calcicludine Incorporating a Disulfide Bond Surrogate

**FIGURE 8:**
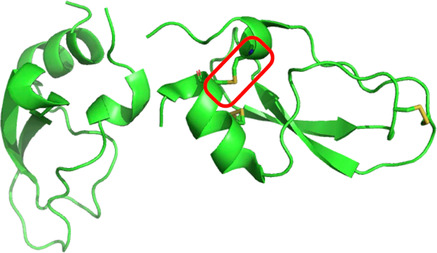
Quasi‐racemic crystal structure of L‐calcicludine which contains a covalent disulfide bond surrogate (highlighted by the red box) with D‐ calcicludine (PDB code 6KZF). [The disulfide bonds of D‐ calcicludine are not shown.]

Native chemical ligation was used for efficient macrocyclization of a calcicludine‐derived long, branched polypeptide chain prepared by chemical synthesis which contained a single covalent disulfide bond surrogate moiety. Subsequent treatment of the surrogate‐constrained macrocyclic polypeptide under standard redox folding conditions formed the remaining disulfides of the calcicludine protein molecule. The structure of the resulting calcicludine analog protein was determined by greatly facilitated quasi‐racemic crystallization with D‐calcicludine protein. X‐ray diffraction data were acquired to a resolution of 2.5 Å and were indexed in space group I41. The structure of the quasi‐racemate was solved by molecular replacement using the NMR structure of calcicludine as search model and showed that the structure of the synthetic calcicludine analog that contained a covalent disulfide bond surrogate was essentially identical to that of native calcicludine (Figure 8) [40].

### 7.14 Racemic Crystallography Used to Verify the Structure and Disulfide Pairings of a Cysteine‐Rich Domain of Tumor Necrosis Factor Receptor 1

#### 7.14.1 TNFR‐1 Cysteine‐Rich Domain 2

Synchrotron diffraction data were collected to a resolution of 1.4 Å and indexed in the centrosymmetric space group P12_1_1. The racemate structure was solved by molecular replacement (PDB code 8P6Q). It confirmed that both enantiomeric forms of the cysteine‐rich domain 2 of TNFR‐1 had preserved the folded structure and disulfide bonds found in the native TNF‐1 protein molecule [41].

### 7.15 Racemic Crystallography of Distantly Related Bacteriocin Proteins

#### 7.15.1 Aureocin A53 and Lacticin Q

These small (51 and 53 amino acid residues) proteins were separately crystallized from racemic mixtures. Synchrotron diffraction data were collected to resolutions of 0.89 and 0.96 Å respectively. The structures of the two bacteriocins were solved by molecular replacement (PDB codes 8AVC and 7P5R) and were compared in order to infer aspects of the molecular basis of their differing anti‐bacterial activities [42].

### 7.16 Quasi‐Racemic Crystallography of a De Novo Designed Protein Molecule

#### 7.16.1 A D‐Protein De Novo‐Designed to Bind an Achiral Drug Molecule

**FIGURE 9:**
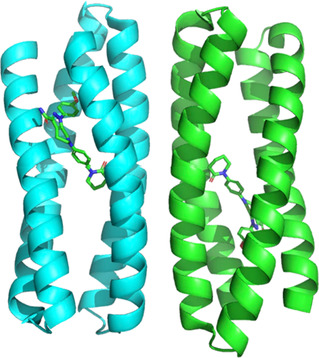
Quasi‐racemate crystal structure of the de novo designed D‐protein (green) bound to the achiral drug molecule apixaban [PDB 8JH6].

A de novo designed 126‐amino acid residue D‐protein prepared by total chemical synthesis was cocrystallized with the recombinantly expressed L‐protein in the presence of an achiral small‐molecule drug to give quasi‐racemic crystals. Diffraction data were collected to a resolution of 1.8 Å and indexed in pseudo‐centrosymmetric space group P1. The structure of the quasi‐racemate, with the achiral small‐molecule drug, was solved by molecular replacement using the crystal structure of the recombinantly expressed L‐protein as search model (Figure 9) [43].

Other protein structures that have been determined by racemic or quasi‐racemic crystallography include ester insulin, human insulin, kaliotoxin, Ts3 toxin, vinillin headpiece domain, and C5a protein. Literature citations to these and other racemic protein structures can be found here [31].

## 8 Summary

The 1995 prediction on theoretical grounds by Wukovitz and Yeates [6] that crystal formation from a racemic protein mixture would be greatly facilitated has been validated in numerous experimental determinations of protein molecular structure by racemic protein X‐ray crystallography. Additionally, it is now abundantly evident that similarly facilitated crystallization is also observed for *quasi*‐racemic mixtures of protein enantiomorphs, mirror image forms of a protein molecule that—while not true enantiomers—are similar enough in overall mirror image folded structures to access highly ordered pseudo‐centrosymmetric crystal packing arrangements (Table 2). Interestingly, the strong preference for centrosymmetric racemic crystal formation is vividly illustrated by the observation that *achiral* cyclic peptoid molecules adopt enantiomeric chiral folded structures in order to crystallize in achiral, centrosymmetric space groups [44, 45].

**TABLE 2:** Racemic and quasi‐racemic protein crystallography.

• Racemic and quasi‐racemic protein mixtures facilitate protein crystallization. • Highly ordered crystals; notable examples of extremely low water content. • Racemic {D‐protein + L‐protein} mixtures crystallize in centrosymmetric space groups. • Quantized phases facilitate structure solution by direct methods. • Higher‐quality electron density maps. • Quasi‐racemic protein mixtures facilitate protein crystallization. • Quasi‐racemic protein mixtures crystallize in pseudo‐centrosymmetric space groups. • Quasi‐racemic mixtures of a {D‐protein + L‐protein analog} facilitate structure determination of protein analogs.

## 9 Discussion

This essay has focused on successful examples of the determination of racemic and quasi‐racemic protein structures reported in the scientific literature. One of the shortcomings of conventional publishing of scientific papers is that failed experiments are rarely published. Even if written up and submitted, failed experiments face huge barriers to acceptance for publication in reputable scientific journals. This is a serious deficiency of the published literature—with rare exceptions, we have no way of knowing what was tried and failed, even though this is potentially valuable information. Racemic and quasi‐racemic protein crystallography are exceptions to the nonreporting of failed experiments. In most of the examples described in this essay, it is reported that even extensive efforts to crystallize the L‐protein alone failed and that racemic or quasi‐racemic mixtures of the same protein molecules readily gave diffraction‐quality crystals. Notably, in a single paper, Wang, Liu, and colleagues reported 10 examples of failure to crystallize L‐protein constructs in extensive trials carried out over months, where in each case quasi‐racemates of the same proteins gave diffraction‐quality crystals within days [34].

Although more limited in number, there are several published examples of failed attempts to obtain crystals from mixtures of racemic or quasi‐racemic protein molecules. Notably, a racemic mixture of the L‐protein and D‐protein enantiomers of human proinsulin failed to give crystals, for unexplained reasons [46]. Insulin quasi‐racemates {human ester insulin + DKP ester insulin} and {human insulin + DKP insulin} also failed to yield crystals of the quasi‐racemates, but had spontaneously resolved to form crystals that contained only human ester insulin or only human insulin. In both those cases, this failure to form crystals of the quasi‐racemate was rationalized as being due to the fact that human insulin and human ester insulin both exist as dimers in solution, while DKP insulin and DKP ester insulin are obligate monomers in solution. The two types of mirror image insulin proteins exist as quite distinct enantiomorphic forms in solution, preventing facile crystal formation [47].

To date, all racemic protein crystals have been centrosymmetric. Consequently, the diffraction data have precise, quantized phases. Determination of the structure of Rv1738 from M. tb illustrated the utility of such quantized phase data: *“Structure refinement also converges faster with* *centrosymmetric data, as the correct phases can be more quickly established early in refinement. This is shown clearly by our test calculations with synthetic data and arises because given a model that is basically correct, even if incomplete, most of the phases will be exactly correct, and refinement can progress in a manner more concerned with fitting the model‐derived amplitudes to the experimental amplitudes than with phase improvement.”* (quoted from [32]). There are other technical advantages to racemic and quasi‐racemic protein crystallography, including a single wavelength phasing strategy discussed here in Ref. [48].

Racemic protein crystallography obligately depends on the preparation of at least one protein enantiomer by total chemical synthesis. To date racemic crystallization has most frequently been applied to small, more readily synthesized protein molecules, typically less than 10 kDa. This can be attributed to the widespread belief that it is difficult or impossible to synthesize protein molecules that are the typical ~30‐kDa size of proteins found in nature. That is a misconception. The ability to make protein molecules by total chemical synthesis was dramatically transformed in the 1990s with the introduction of chemical ligation, the condensation of *unprotected* peptide segments. This innovation rendered straightforward the chemical synthesis of proteins and protein domains containing ~150 amino acid residues, and the product polypeptide chains of that size can be further condensed in straightforward fashion, to give synthetic protein molecules up to ~30‐kDa size [12]. It should be emphasized that the D‐protein molecule used to facilitate crystallization of an L‐protein (or, vice versa) *does not need to be a perfect* *enantiomer of the recalcitrant protein molecule; it simply needs to be a reasonable enantiomorph of the L‐protein molecule.* This fact can be used to greatly simplify the chemical synthesis of suitable D‐protein constructs [36, 37].

Since the first report of greatly facilitated crystallization of a *quasi*‐racemic protein pair [22], the use of quasi‐racemic protein mixtures for X‐ray crystallography has been repeatedly validated. To apply quasi‐racemic protein crystallography to larger proteins or protein complexes, one should take advantage of the ability of independently synthesized protein domains to self‐assemble into functional protein molecules [17], and the demonstrated utility of monomer‐oligomer quasi‐racemic protein crystallography [37]. The straightforward determination of the racemic crystal structure of a heterochiral protein complex [29],in which multiple enantiomers of different protein molecules sorted themselves into a defined quaternary racemate, suggests that individual D‐protein copies of the different domains could be used to facilitate the determination of the crystal structures of large, multi‐domain L‐protein molecules as suggested by Wang, Liu, and colleagues [37]. The multi‐domain L‐protein used for structure determination could itself consist of either a contiguous full‐length polypeptide chain, or of individually synthesized L‐protein domains. Similarly, synthetic L‐neoglycan‐D‐protein enantiomorphs could be used for quasi‐racemic crystallography of glycoproteins with the objective of facilitating the determination of the glycoprotein crystal structure including the complete structures of the glycan moieties. Simply put, the goal is to *facilitate* protein structure determination.

A survey of the very recent literature (2023–2025) suggests that there has been a marked decrease in the number of papers that make use of racemic and/or quasi‐racemic protein crystallography. It is to be hoped that the experimental determination of the structures of protein molecules and protein complexes by X‐ray crystallography will continue to be performed, even in the era of high‐fidelity protein structure prediction [49]. AlphaFold [5] and related deep‐learning‐based protein structure prediction methods are empirical statistical models trained on large datasets of experimentally determined protein molecular structures [1]. These models infer the language correlating amino acid sequence with folded protein structures, often with great accuracy, but their predictions are not substitutes for experimentally determined protein structures [50].

## Conflicts of Interest

The author declares no conflicts of interest.

## Endnotes

1
A space group describes the way the molecules are arranged within the three‐dimensional crystal lattice.

2
An object is said to be “chiral” if cannot be perfectly superimposed on its own mirror image.

3
In a “centrosymmetric” space group crystal lattice, enantiomers are arranged in pairs as if the molecule were reflected through a single point, the inversion center.

4
“Domains” are self‐folding regions of a protein molecule's polypeptide chain.

5
An amusing anecdotal note: early crystal structures of individual mirror image D‐protein molecules (i.e., not racemic structures) submitted to the Protein Data Bank (PDB) were invariably “corrected” by the data checkers to be displayed as L‐protein molecules. Natural proteins are of L‐chirality, so data checkers “corrected” the D‐protein structures to L‐proteins!

6
The following paragraphs describing racemic crystallography of sfAFP are adapted from Ref. [22] describing the work carried out in the Author's laboratory.

7
Note that for true centrosymmetric structures, such as a protein racemate (pair of enantiomers), Friedel's law holds even in the presence of an anomalous scattering atom and it is not possible to measure anomalous differences; hence, MAD phasing cannot be used [51].

8
IUPAC defines quaternary protein structure as “The defined organization of two or more macromolecules with tertiary structure such as proteins that are held together by hydrogen bonds and van der Waals and coulombic forces” [52].
